# The first *Cordyla* Meigen species (Diptera, Mycetophilidae) from continental Australia and Tasmania

**DOI:** 10.3897/zookeys.342.6045

**Published:** 2013-10-14

**Authors:** Olavi Kurina, Sarah Siqueira Oliveira

**Affiliations:** 1 Institute of Agricultural and Environmental Sciences, Estonian University of Life Sciences, Kreutzwaldi st 5D, 51014 Tartu, ESTONIA; 2 Departamento de Biologia, Faculdade de Filosofia, Ciências e Letras de Ribeirão Preto, Universidade de São Paulo, Av. Bandeirantes 3900, 14.040–901 Ribeirão Preto SP, BRAZIL

**Keywords:** Diptera, Mycetophilidae, *Cordyla*, new species, Australia, Tasmania

## Abstract

A new species of Mycetophilidae, *Cordyla australica* **sp. n.**, is described from continental Australia and Tasmania, representing the first *Cordyla* record in the region. A detailed description of its morphology with illustrations of male and female terminalia and a map of the collecting localities are provided. According to the structure of male terminalia, *Cordyla australica* **sp. n.** belongs to the *Cordyla murina* species-group that has 13 species worldwide. Within the group *Cordyla australica* **sp. n.** resembles *Cordyla murina* but has a unique outline of the hypoproct and medial branch of the gonostylus. The observed distributional pattern is restricted to the rainforest of eastern Australia and Tasmania.

## Introduction

The genus *Cordyla* Meigen, 1803, a member of the tribe Exechiini of Mycetophilidae, is a well delimited monophyletic clade of fungus gnats (Diptera: Sciaroidea). Having been treated earlier also in Mycetophilini (since Edwards 1925), the genus was transferred to Exechiini by Tuomikoski (1966). Within the tribe, *Cordyla* has a rather isolated position forming by Rindal et al. (2009) a common clade with *Brachypeza* Tuomikoski. However, the recently described genus *Brachyradia* Ševčík & Kjærandsen including two species from the Oriental and Australasian (Papua New Guinea) regions shares several synapomorphies with *Cordyla*, and thus may be the closest relative instead of *Brachypeza* (cf. Ševčík and Kjærandsen 2012). The *Cordyla* species are characterized mainly by short antennae with reduced number of flagellomeres and swollen antepenultimate palpal segment (Tuomikoski 1966) while by characters in male terminalia, the species are divided into three subgeneric groups (Kurina 2001). *Cordyla* specimens are easily recognisable by small size, humpbacked habitus in combination with mainly dark coloration and, especially, by swollen antepenultimate segments of palpi.

Thirty-eight described *Cordyla* species are known worldwide at present, viz. twenty-four from the Palaearctic region (Kurina 2005 and references therein, Sasakawa 2008), ten from the Nearctic region (Bechev 2000), three from the Oriental region (Ševčík 2001, Kurina 2005) and one from the Autralasian region: Northeastern Papua New Guinea (Kurina 2005). The genus is also known from undescribed species from the Neotropical region (Colombia (Oliveira et al. 2007) and Central America (Vockeroth 2009, OK and SSO *pers. obs.*)). There are no published records of *Cordyla* species from Afrotropical region.

The aim of this paper is to describe and illustrate the first *Cordyla* species from continental Australia and Tasmania and discuss its systematics.

## Material and methods

The material was collected from seven localities in Tasmania using mostly Malaise traps, in few cases also pitfall traps or sweeping. A good amount of material comes from the Warra Long-Term Ecological Research Site (for details see Brown et al. 2001). In the Australian continent, the material was collected: 1) from Carrai and Werrikimbe Plateaus (both in NSW) during the tree trunk invertebrate survey by sticky traps (for details see Bickel and Tasker 2004); 2) from Brisbane Forest Park by Malaise traps and 3) from Victoria, Coopracambra National Park by Malaise traps. For collecting localities see Fig. 15. All specimens were stored initially in ethyl alcohol within which most of them – after studying under a stereomicroscope Leica S8APO or Leica MZ16 – are still preserved. In case of several specimens, for more detailed study of male terminalia, they were detached and macerated in a solution of KOH, followed by neutralization in acetic acid and washing in distilled water. The remaining chitinous parts were thereafter inserted into glycerine for study including illustrations and preserved as glycerine preparations in polyethylene microvials (cf. Kurina 2003). A few specimens including their terminalia were slide mounted in Euparal following the method described by Kurina (2008). The holotype was mounted from alcohol, using a chemical method described by Vockeroth (1966), and double-pinned. The preservation method of each specimen is indicated in the material section. The measurements are given as the range of measured specimens followed by the mean value. While not otherwise stated, five specimens were measured, while the measurements and setosity information from the holotype are given in square brackets. The ratios of the three apical palpal segments are given as 3^rd^:4^th^:5^th^. All measurements are taken from specimens in alcohol. Morphological terminology follows generally that of Søli et al. (2000) while the interpretation by Kjærandsen (2006) and Oliveira and Amorim (2012) are used for terminalia and thorax, respectively.

The habitus photo has been made in alcohol medium using the Canon 7D camera in combination with Canon MP-E65 (F2.8 1–5×) lens (see Kurina et al. 2011). The photos of terminalia were combined by software LAS V.4.1.0. from multiple gradually focused images taken by a camera Leica DFC 450 attached to the compound microscope Leica DM 6000 B. Adobe Photoshop CS5 was used for enhancing the figures and compiling the plate. The map was done plotting the coordinates at the Google website and then edited in the Adobe Photoshop 8.0.1.

The following acronyms are used for depositories:

AMSA Australian Museum, Sydney, Australia

ANIC Australian National Insect Collection, Canberra, Australia

IZBE Institute of Agricultural and Environmental Sciences, Estonian University of Life Sciences [former Institute of Zoology and Botany], Tartu, Estonia

MZUSP Museu de Zoologia da Universidade de São Paulo, Brazil

SMNH Swedish Museum of Natural History, Stockholm, Sweden

TFIC Tasmanian Forest Insect Collection, Hobart, Australia

## Data resources

Specimen information is available for download in Darwin Core 1.4 format at GBIF, the Global Biodiversity Information Facility, http://ipt.pensoft.net/ipt/resource.do?r=cordyla.

## The species

### Cordyla australica sp. n.

http://zoobank.org/22F4AE20-D2B3-42EE-9089-0A29BC3E9ECF

http://species-id.net/wiki/Cordyla_australica

Figs 1 –15

#### Type material.

*Holotype*. 1♂, AUSTRALIA: Tasmania, Warra LTER: Manuka Road, Malaise trap, 43.07°S, 146.67°E, 20.iv.2004, R. Bashford leg., plot code: SSTSMA254, sample code FT30575 (mounted from alcohol, in AMSA).

*Paratypes*. 5♂♂, same as holotype (in alcohol, 2 in TFIC, 3 in IZBE); 8♂♂, same as holotype except 19.vii.2004, plot code: SSTSMA254, sample code: FT30660 (in alcohol, 3 in AMSA, 5 in IZBE); 15♂♂ 2♀♀, same as holotype except 1.vii.2005, plot code: SSTMID280 and sample code: FT36772 (in alcohol, in IZBE); 2♂♂, same as holotype except 1.vii.2005 and plot code: SSTEAS094, sample code FT36767 (in alcohol, in TFIC); 1♂, same as holotype except 2.v.2003, plot code: SSTTOP060, sample code FT29026 (in alcohol, in TFIC); 1♂, same as holotype except 1.iii.2005, plot code: SSTEAS318, sample code FT35684 (in alcohol, in IZBE); 2♂♂, same as holotype except 13.x.2002, plot code: SSTSMA254, sample code FT28944 (in alcohol, in IZBE); 3♂♂, same as holotype except 19.v.2004, plot code: SSTCON059, sample code FT30632 (in alcohol, in IZBE); 1♂, same as holotype except 19.v.2004, plot code: SSTTOP060, sample code: FT30634 (in alcohol, in IZBE); 1♂, same as holotype except 13.x.2002, plot code: SSTMID160, sample code FT7034 (in alcohol, in IZBE); 2♂♂, same as holotype except 1.iv.2005, plot code: SSTWES120, sample code FT35962 (in alcohol, in IZBE); 1♂, same as holotype except 2.iv.2007, plot code: SSTCON059, sample code FT40220 (in alcohol, in IZBE); 1♂, same as holotype except 3.ix.2007, plot code: SSTCON059, sample code FT40745 (in alcohol, in IZBE); 1♂, same as holotype except 5.iii.2007, plot code: SSTCON059, sample code FT40115 (in alcohol, in IZBE); 1♂ same as holotype except 24.iii.2000, plot code: SSTSMA663, sample code FT28616 (in alcohol, in IZBE); 2♂♂, Tasmania, Mount Warra - Mt. Weld alt. Transect 100, Malaise trap, 43.07S, 146.67E, 27.ii.2002, N. Doran & R. Bashford leg., plot code WR0100M, sample code FT5923 (in alcohol, in IZBE); 1♂, same as previous except 27.ii.2001, sample code: FT19 (in alcohol, in IZBE); 6♂♂, same as previous except 27.iv.2001, sample code: FT199 (in alcohol, in IZBE);1♂, same as previous except 27.ii.2001, plot code: WR0200M, sample code: FT26 (in alcohol, in IZBE); 3♂♂, AUSTRALIA, Tasmania, Ewart creek, 150m dstr bridge on A10, 221 m.a.s.l., Malaise trap, loc 12, 41°58.576'S, 145°27.708'E, 22.ii–03.iii.2006, Jönsson, N., Malm, T. & Williams, D. leg. (on slides, in SMNH); 23♂ 1♀ AUSTRALIA, Tasmania, Ewart creek, Malaise trap ethanol, 41°58'S, 145°28'E, 16.i–02.ii.1983, I.D. Naumann & J.C. Cardale leg. (1♂ 1♀ on slides, in MZUSP; 22♂♂ in alcohol, 5♂♂ in MZUSP other in ANIC); 23♂♂ 2♀♀, AUSTRALIA, Tasmania, Central Plateau, small creek flowing into Arthur’s Lake, 50m dst 1^st^ bridge on gravel road from Rd B51 to Little Lake, 1006 m.a.s.l., Malaise trap, loc 17, 41°57.237'S, 146°51.928'E, 25.ii–04.iii.2006, Jönsson, N., Malm, T. & Williams, D. leg. (1♂ 1♀ on slides other in alcohol, 3♂♂ in IZBE other in SMNH); 5 ♂♂, AUSTRALIA, Tasmania, Cradle MTN NP. creek from Crater Lake to Ronny Greek 100m upstr broadwalk, 867 m.a.s.l., Malaise trap, loc 14, 41°38.667'S, 145°56.775'E, 23.ii–04.iii. 2006, Jönsson, N., Malm, T. & Williams, D. leg. (1♂ on slide other in alcohol, in SMNH); 1♂, AUSTRALIA, Tasmania, Southwest National Park, in forest 20m off Rd C607, 300m south off Creepy Crawly Walk, 573 m.a.s.l., Malaise trap, loc 9, 42°50.012'S, 146°22.866'E, 21.ii.–01.iii.2006, Jönsson, N., Malm, T. & Williams, D. leg. (in alcohol, in SMNH); 5♂♂, AUSTRALIA: Queensland, Brisbane Forest Park, Enoggera Creek at Scrub Road crossing, in tropical rain forest with *Eucalypus* spp., Malaise trap, 27°25'42"S, 152°50'33"E, 14–29.xi.1995, 1–7.xii.1995, 7–27.xii. 1995 and 28.xii.1995–4.i.1996, Irwin, M.E. leg. (in alcohol, 3 in ANIC, 2 in IZBE).

#### Other material studied

(not included in paratypes due to quality of material from sticky traps). 1♀, AUSTRALIA: New South Wales, Werrikimbe National Park, 31°16'50"S, 152°03'19"E, 1045m, sticky trap on *Eucalypus saligna*, 1.xii–7.xii.1997, E. Tasker leg., WS-FC-127-6 (K377114, in alcohol, in AMSA); 1♀, AUSTRALIA: New South Wales, Werrikimbe National Park, 31°10'23"S, 152°09'45"E, 1060m, sticky trap on *Eucalypus saligna*, 1.xii–7.xii.1997, E. Tasker leg., WS-KF-127-6 (K377115, in alcohol, in AMSA); 1♀, AUSTRALIA: New South Wales, Carrai State Forest, 30°58'48"S, 152°17'06"E, 975m, sticky trap on *Eucalypus obiqua*, 11–16.i.1998, E. Tasker leg., CR-RO-018-4 (K377116, in alcohol, in AMSA); 1♀, AUSTRALIA: New South Wales, Werrikimbe National Park, 31°16'50"S, 152°03'19"E, 1045m, sticky trap on *Eucalypus campanulata*, 1.xii–7.xii.1997, E. Tasker leg., WS-FC-127-3 (K377117, in alcohol, in AMSA); 2♀♀, AUSTRALIA: New South Wales, Carrai State Forest, 30°59'45"S, 152°16'23"E, 930m, sticky trap on *Eucalypus campanulata*, 3.xii–8.xii.1997, E. Tasker leg., CS-FZ-127-4 (K377118, in alcohol, in AMSA); 1♂1♀, AUSTRALIA: New South Wales, Carrai State Forest, 30°59'45"S, 152°16'23"E, 930m, sticky trap on *Eucalypus saligna*, 3.xii–8.xii.1997, E. Tasker leg., CS-FZ-127-5 (K377119, in alcohol, in AMSA); 1♂, AUSTRALIA: New South Wales, Carrai State Forest, 30°54'19"S, 152°17'36"E, 1055m, sticky trap on *Eucalypus campanulata*, 3.xii–8.xii.1997, E. Tasker leg., CC-DP-127-4 (K377120, in alcohol, in AMSA); 1♀, AUSTRALIA: New South Wales, Carrai State Forest, 30°54'35"S, 152°16'26"E, 1090m, sticky trap on *Eucalypus obiqua*, 3.xii–8.xii.1997, E. Tasker leg., CC-FK-127-3 (K377121, in alcohol, in AMSA); 4♂♂, AUSTRALIA: New South Wales, Werrikimbe National Park, 31°16'50"S, 152°03'19"E, 1045m, sticky trap on *Eucalypus obiqua*, 3.vii–8.vii.1998, E. Tasker leg. WS-FC-078-1 (K377122, 1♂ in slide, 3♂♂ in alcohol, in AMSA); 1♀, AUSTRALIA: New South Wales, Carrai State Forest, Feltons Knob, 30.9097S, 152.2739E; 1090m, 24.iv–30.iv.1998, E. Tasker, P. German leg., CC-FK-048-3 (K377123, in alcohol, in AMSA); 2♂♂1♀, AUSTRALIA: New South Wales, Werrikimbe National Park, 31°16'50"S, 152°03'19"E, 1045m, sticky trap on *Eucalypus obiqua*, 3.vii–8.vii.1998, E. Tasker leg., WS-FC-078-3 (K377124, in alcohol, in AMSA); 1♂1♀, AUSTRALIA: New South Wales, Carrai State Forest, 30°54'33"S, 152°16'28"E, 1075m, sticky trap on *Eucalypus campanulata*, 3.xii–8.xii.1997, E. Tasker leg., CC-CR-127-2 (K377125, in alcohol, in AMSA); 2♂♂, AUSTRALIA: Tasmania, King William Creek Site, 43 08 84E, 5 22 76 00N [these label data are unclear, the approximate geographic coordinates are 42°12'S, 146°8'24"E], pitfall, 23.ii.2000, M. Driessen leg. (K377126 and K377128, in alcohol, in AMSA); 1♂, AUSTRALIA: Tasmania, Lake St Clair, Site: SCRW, sweep, 28.viii.1999, (K377127, in alcohol, in AMSA); 43♂♂, AUSTRALIA: Victoria, Coopracambra National Park, Beehive creek, 27 Km NNE Cann R., 347 m, Malaise traps, 37°20'01"S, 149°14'12"E, 5.xii.2004–12.i.2005, C. Lambkin & N. Starick leg., ANIC sample 2608 (material from ANIC in donation to SSO, housed at the Universidade de São Paulo, campus of Ribeirão Preto).

#### Description.

**Figures 1–3.**
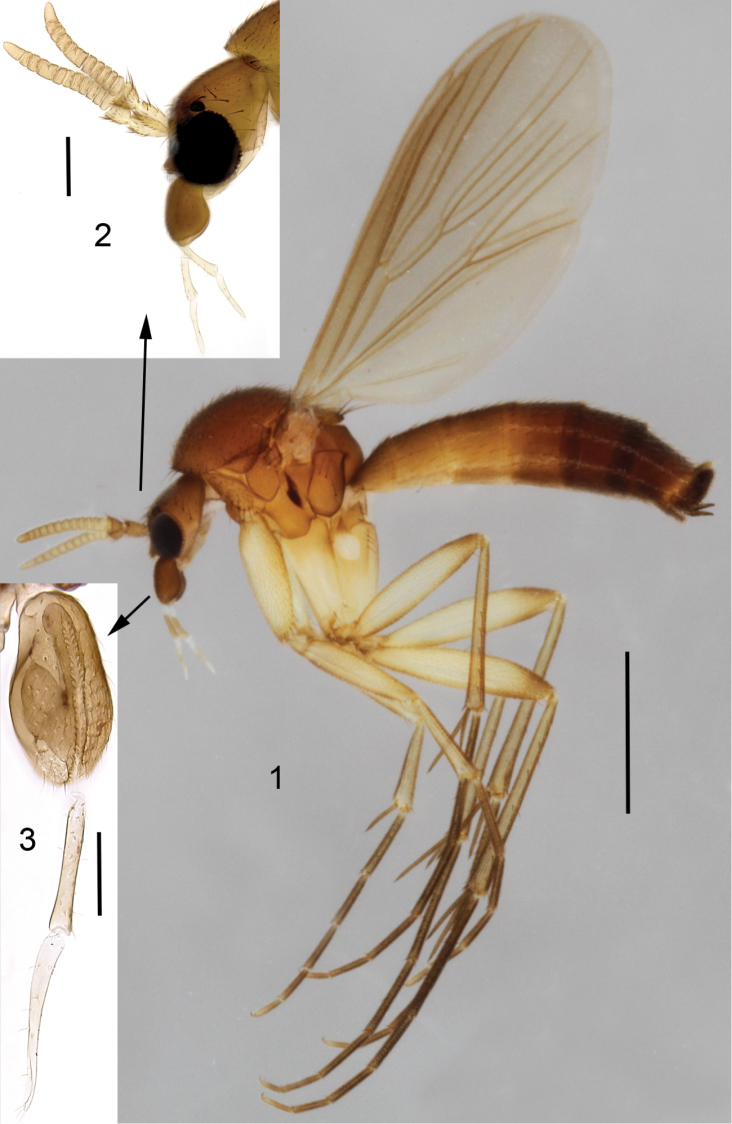
*Cordyla australica* sp. n. **1** male habitus **2** head with antennae and maxillary palpi, closer view **3** three apical segments of maxillary palpus. Scale bar = 1 mm (**1**), 0.2 mm (**2**) and 0.1 mm (**3**).

**Male** (Fig. 1). Total length 2.4–3.7, 2.9 [3.1] mm (n=10).

**Figures 4–9.**
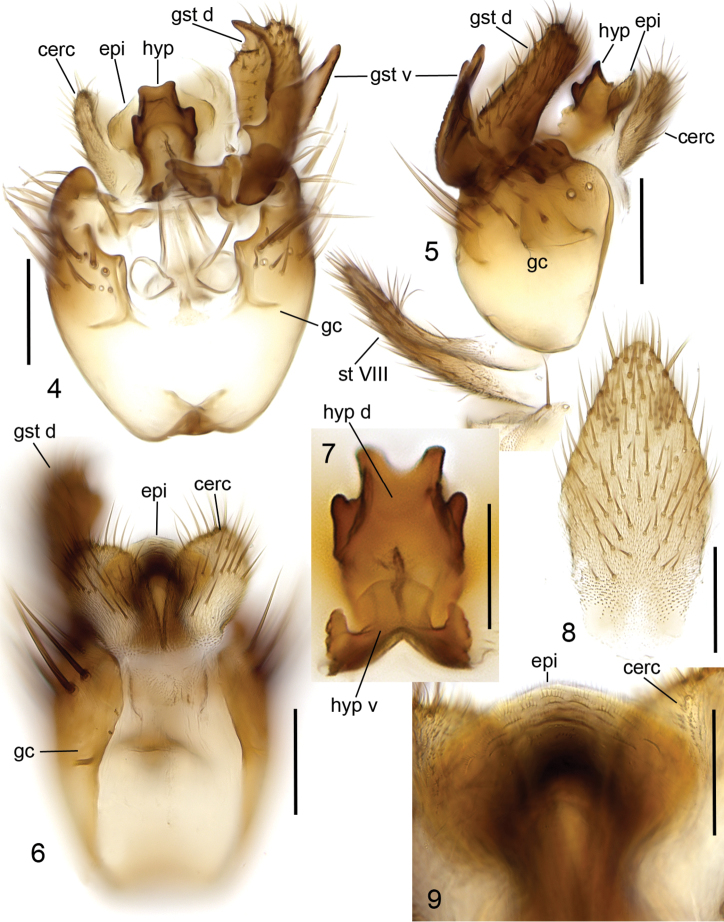
*Cordyla australica* sp. n., male terminalia. **4** ventral view **5** lateral view **6** dorsal view **7** hypoproct, ventral view **8** sternite VIII, ventral view **9** epiproct, dorsal view. Scale bar 0.1 mm (**4, 5, 6, 8**) and 0.05 mm (**7, 9**). Abbreviations: cerc = cercus; epi = epiproct; gc = gonocoxite; gst d = dorsal branch of gonostylus; gst m = medial branch of gonostylus; gst v = ventral branch of gonostylus; hyp = hypoproct; st VIII = sternite VIII.

**Figures 10–11.**
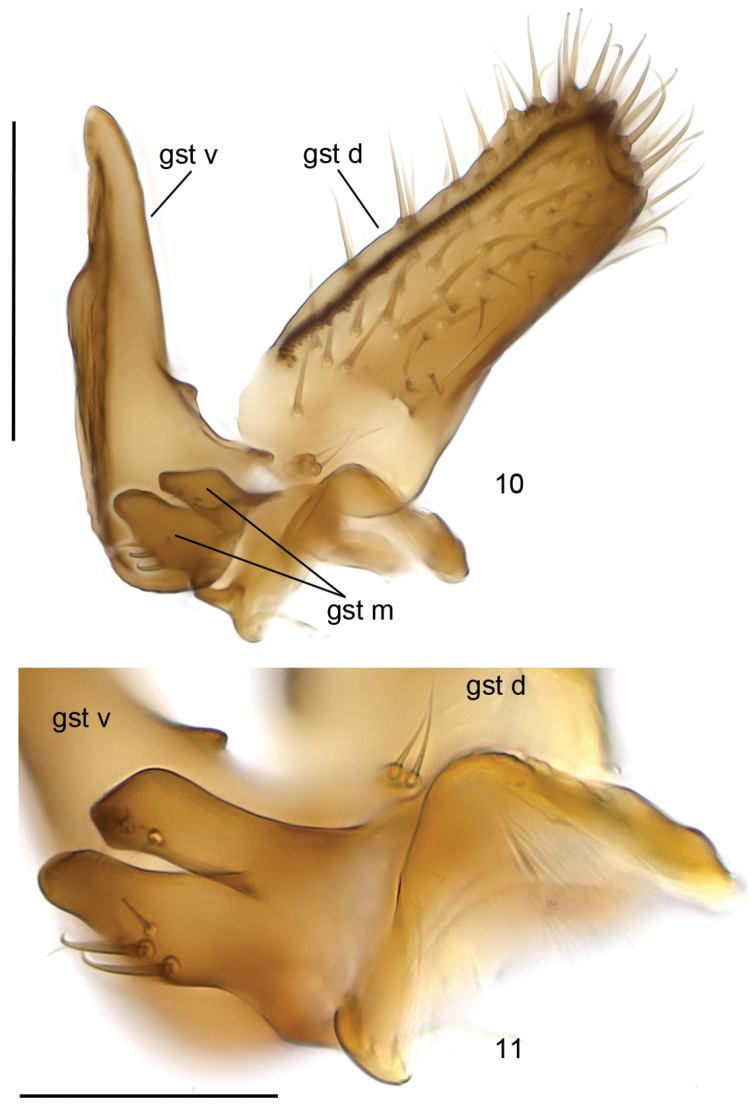
*Cordyla australica* sp. n., gonostylus. **10** internal view **11** lobes of medial branch of gonostylus. Scale bar = 0.1 mm (**10**) and 0.05 mm (**11**).

**Head** (Fig. 2) brown, mouthparts yellowish. Two ocelli encircled by dark brown areas, close to compound eyes. All three visible palpal segments (Fig. 3) setose, swollen antepenultimate segment blackish brown, succeeding segments light brown, basally pale. 4^th^ segment slightly widening apically, 5^th^ segment apically tapering. Swollen palpal segment 1.7–2.1, 1.9 [2.1] times as long as broad medially from lateral view, and 1.0–1.2, 1.1 [1.1] times as long as height of compound eye. Ratios of three apical palpomeres 1.0: 0.8–0.9, 0.8 [0.9]: 0.9–1.0, 1.0 [0.9]. Antenna light brown with 2+12 segments. Scape and pedicel with brown setae, flagellum with somewhat paler setosity. Scape elongate cup-shaped, 2.0–2.3, 2.1 [2.0] times as long as wide apically. Pedicel cup-shaped, 0.7–0.8, 0.8 [0.7] times as long as wide apically. Flagellomeres rectangular, about twice as wide as long. Apical flagellomere conical, about 1.6 times as long as wide basally. **Thorax** brown, mesonotum and hind margin of laterodergite somewhat darker. Anterior part of mesepimeron with a blackish patch leaving anteroapical corner light brown. Haltere with pale knob, stem basally pale and apically brown. All setosity on thorax brown. Scutum entirely covered with decumbent setae, scutellum with setae including two pairs of marginal bristles, laterals considerably shorter than internals. Antepronotum with setae including 4-5 [4] bristles, proepisternum with setae including 6–8 [8] bristles. Anepisternum with 4–6 [6] bristles at hind margin and with ca. 40 setae on its upper two third. Mesepimeron and katepisternum bare. Laterotergite with 4 bristles and ca. 10 setae. Mediotergite bare. Metepisternum with 3–5 [5] bristles and ca. 10 setae. **Wing** with yellowish tinge, otherwise clear. Length 1.9–2.8, 2.3 [2.6] mm (n=10). Ratio of length to width 2.5–2.8, 2.6 [2.5]. All veins light brown. Radial veins seem darker because of setae on both surface; other veins bare. Crossvein r-m apically disjunct. M-stem about 4 times as long as r-m. R5 slightly sinusoid. M2 not reaching wing margin, broken 0.8–1.2, 1.0 [0.9] times of m-stem length before it. Cu-fork begins very slightly before medial fork. **Legs** yellow, with fore-femur infuscated ventrally and mid- and hind femurs infuscated at apical fifth. Tarsi seem darker because of dense brown setae. Hind coxa with 4–5 [5] posteroleteral bristles basally, with one lateral and one posterior bristle apically, and with ca. 25 weaker setae along posterolateral margin. Ratio of femur to tibia for fore-, mid- and hind legs: 1.3–1.4, 1.4 [1.4]; 1.0, 1.0 [1.0]; 0.9–1.0, 0.9 [0.9]. Ratio tibia to first tarsomere for fore-, mid- and hind legs: 1.1–1.2, 1.2 [1.1]; 1.2, 1.2 [1.2]; 1.4–1.5, 1.5 [1.5]. Fore-tibia with a spur about 0.5–0.6, 0.5 [0.6] of fore basitarsus; mid-tibia with anterior spur about 0.3–0.4, 0.4 [0.3] and with posterior spur about 0.6–0.7, 0.6 [0.6] of mid basitarsus; hind tibia with anterior spur about 0.5– 0.6, 0.5 [0.6] and with posterior spur about 0.6–0.7, 0.6 [0.6] of mid basitarsus. **Abdomen** with 3 or 4 segments dorsally brown, laterally and ventrally yellow; succeeding segments brown to dark brown. **Terminalia** (Figs 4-11) two-coloured: basal part of gonocoxite and cerci yellow; apical part of gonocoxite and gonostylus brown; sternite 8 seems brownish because of dense setosity. Sternite 8 ovate with bluntly rounded apex, basal quarter membranous and bare, setae on apical quarter somewhat stronger than rest of them. Gonocoxite slightly oblong, with broad ventral incision about half of gonocoxite height. Dorsal medial margin of gonocoxite bulging mesiad at apical third. Cerci setose, basally membranous and fused, apically rounded, protruding well over gonocoxite. Ventral margin of gonocoxite angular. Basal half of gonocoxite bare, apical half with strong bristles. Dorsal branch of gonostylus rectangular, apically drawn into a pointed lobe, with a sclerotized comb on its ventral surface, as long as branch height. Setosity homogeneous without any deviations. Dorsal branch of gonostylus with a basal tubercle on its ventral surface close to base of medial branch; tubercle with two apical setae. Ventral branch of gonostylus bare, subequal to dorsal branch, with serrated lateral margin and with a hump on basal third of medial margin. The apical third of ventral branch is well tapering in ventral view. Medial branch of gonostylus divided at apical two third into two subequal lobes: ventral lobe apically rounded, medially somewhat swollen, slightly curved dorsad, with three setae on its ventral margin medially; dorsal lobe apically angular with two setae on apical third. Epiproct campaniform with small setulae that arise in lines of 4 to 8 from small ridges. Hypoproct consists of basally connected dorsal and ventral parts: both parts are with well-outlined lateral shoulders, the dorsal part is apically notched while the ventral part is apically convex.

**Figures 12–14.**
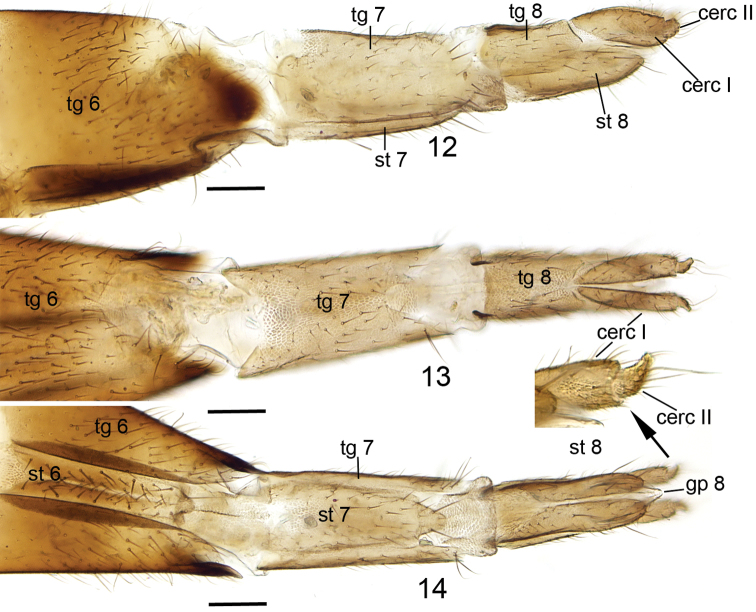
*Cordyla australica* sp. n. female terminalia. **12** lateral view **13** dorsal view **14** ventral view. Scale bar = 0.1 mm. Abbreviations: cerc= cercus, gp= gonapophysis, st= sternite, tg= tergite.

**Female.** Total length 2.2–3.4, 3.0 mm. Wing length 1.6–2.8, 2.2 mm. Ratio of length to width 2.5–2.9, 2.7. Antennae 2+9 segments. By setosity and coloration similar to male, except for entirely light brown abdomen in some specimen. Terminalia (Figs 12-14) light brown. Cercus two-segmented: apical segment small, apically tapering and bent laterad in ventral and dorsal views, with 2-3 long setae deviating from other setosity; basal segment long ovate, slightly sinusoidal and wider than apical segment. Gonapophysis VIII membranous, visible in ventral view, apically somewhat pointed. Tergite VIII rectangular, subequal to length of basal segment of cercus, apically angular, basally emarginated in dorsal view. Sternite VIII lateroapically conical, with deep ventral cleft. Tergite VII about twice as long as tergite VIII, with basal and apical broad incision dorsally and with a few apical stronger setae deviating from other setosity. Sternite VII apically conical, subequal to length of tergite VII. Tergite VI with conical and sclerotized apical edge laterally and with broad incision apicodorsally.

#### Biology.

Unknown.

#### Etymology.

The species is named to indicate its discovery in Australia.

#### Comments.

The species shows high variation (up to 35%) in body size that is, however, continuous and observed also in other *Cordyla* species (e.g. in European *Cordyla crassicornis* Meigen, 1818: OK *pers. obs.*) and colour variation including some specimens darker than others. Despite of that, we have not found any species level morphological differences within the studied material.

## Discussion

According to structure of male terminalia, *Cordyla australica* sp. n. belongs to the *Cordyla murina* species-group as outlined by Kurina (2001). The species of this group have gonostylus with medial branch divided into two lobes of which outline and setosity are species-specific. Thirteen species belong to the group as follows: seven and five in the Palaearctic and Nearctic region, respectively and one from the Australasian region. Within the group, in respect to the number of flagellomeres – an important character for species grouping since Landrock (1926) – males of *Cordyla australica* sp. n. shares 12 flagellomeres with 4 species, viz. *Cordyla murina* Winnertz, 1863 (widely in Palaearctic), *Cordyla styliforceps* (Bukowski, 1934) (southern Europe), *Cordyla bidenticulata* Sasakawa, 2003 (Japan) and *Cordyla toraia* Kurina, 2005 (South Sulawesi and Papua New Guinea). Having antepenultimate palpal segment dark brown to blackish, ventral branch of gonostylus with one serrated margin and notched dorsal part of hypoproct *Cordyla australica* sp. n. is most similar to *Cordyla murina*. However, the dorsal part of hypoproct is deeply notched and without lateral shoulders in *Cordyla murina*, while the notch is shallow and lateral shoulders are well developed in *Cordyla australica*. In the ventral part of the hypoproct, *Cordyla murina* has lateral shoulders protruding over the slightly convex medial area, while the latter is well convex with subequal lateral shoulders in *Cordyla australica* sp. n. *Cordyla murina* has the lobes of medial branch of gonostylus apically slightly swollen and billed, while they have a different outline in *Cordyla australica* (cf. Fig. 11). According to the key of Oriental and Australasian species (Kurina 2005), *Cordyla australica* runs to the couplet 2 (*Cordyla toraia*) but differing by the dark brown antepenultimate palpal segment (yellow in *Cordyla toraia*), outline of hypoproct (cf. Fig. 7 and Kurina 2005: fig. 11) and other details in male terminalia.

**Figure 15.**
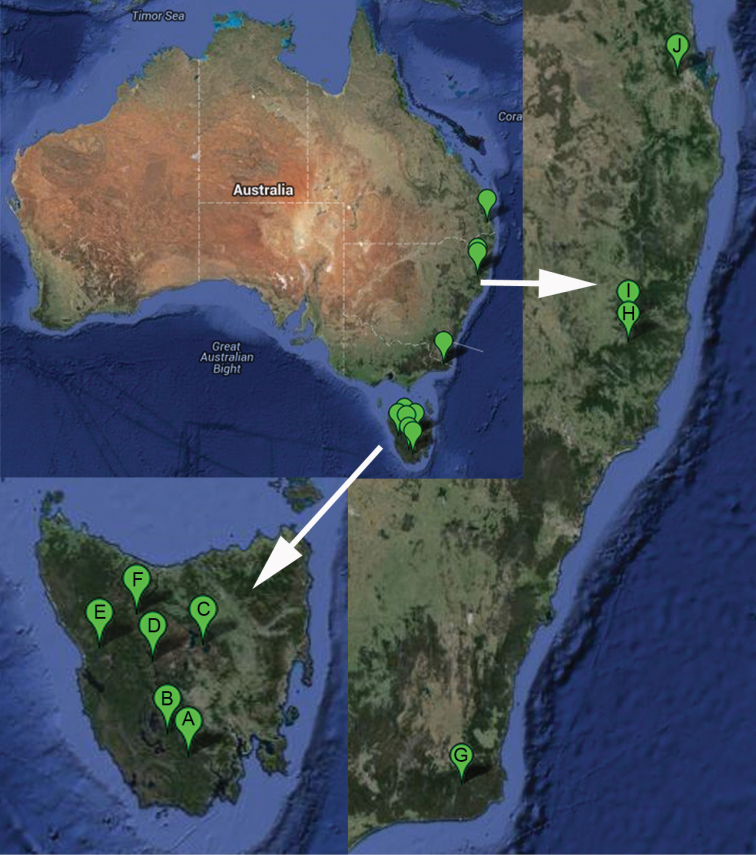
Collecting localities of *Cordyla australica* sp. n. in the continental Australia and Tasmania. **A** Tasmania, Warra long-term ecological research site **B** Tasmania, Southwest National Park **C** Tasmania, Central Plateau **D** Tasmania, King William Creek **E** Tasmania, Ewart creek **F** Tasmania, Cradle Mountain **G** Victoria, Coopracambra National Park **H** New South Wales, Werrikimbe National Park **I** New South Wales, Carrai State Forest **J** Queensland, Brisbane Forest Park.

In spite of wide range of the studied Australian samples (SSO *pers. obs.*), *Cordyla australica* sp. n. is apparently found only in wet forest of eastern Australia and Tasmania (Fig. 15).

## Supplementary Material

XML Treatment for Cordyla australica

## Appendix

Occurence data of Australian *Cordyla*. (doi: 10.3897/zookeys.342.6045.app) File format: Mircosoft Excel file (xls).

**Explanation note:** Occurence data of a new species – *Cordyla australica* Kurina & Oliveira, 2013 – based on material from Austarlian mainland (Victoria, New South Wales, Queensland) and Tasmania are provided.
